# Active Control of a Small-Scale Wind Turbine Blade Containing Magnetorheological Fluid

**DOI:** 10.3390/mi9020080

**Published:** 2018-02-14

**Authors:** Fevzi Cakmak Bolat, Selim Sivrioglu

**Affiliations:** 1 Department of Mechanical Engineering, Bayburt University, 69000 Bayburt, Turkey; fevzibolat@bayburt.edu.tr; 2 Department of Mechanical Engineering, Gebze Technical University, 41400 Kocaeli, Turkey

**Keywords:** active vibration control, elastic blade, magnetorheological fluid, robust control

## Abstract

This research study proposes a new active control structure to suppress vibrations of a small-scale wind turbine blade filled with magnetorheological (MR) fluid and actuated by an electromagnet. The aluminum blade structure is manufactured using the SH3055 (Bergey Windpower Co. Inc., Norman, OK, USA) code numbered airfoil which is designed for use on small wind turbines. A dynamic interaction model between the MR fluid and the electromagnetic actuator is constructed to obtain a force relation. A detailed characterization study is presented for the proposed actuator to understand the nonlinear behavior of the electromagnetic force. A norm based multi-objective H_2_/H_∞_ controller is designed using the model of the elastic blade element. The H_2_/H_∞_ controller is experimentally implemented under the impact and steady state aerodynamic load conditions. The results of experiments show that the MR fluid- electromagnetic actuator is effective for suppressing vibrations of the blade structure.

## 1. Introduction

The engineering flexible structures like beams, plates and shells show modal dynamical behavior with external disturbances. It is a desired characteristic for such engineering structures to have adaptive behavior to changing external conditions. The control of adaptive structures also affects the dynamics of the flexible systems. Active vibration control approaches improve the performance in terms of reduction in vibration amplitude of the structures compared with passive systems. Actively controlled flexible structures with piezoelectric (PZT) material have been studied by many researchers to create smart or adaptive systems. Although PZT actuators have great potentials especially for aerospace applications, there are some difficulties in realization for large engineering systems due to limited outputs of PZTs. 

In recent years, magnetorheological (MR) materials have been attracted much attention for use in engineering systems [1,2,3]. MR fluids contain ferromagnetic particles that can change its rheological properties under application of an external magnetic field. The MR fluid has distinct properties for engineering applications and can be used in vibration control studies [4,5,6,7,8,9,10]. Basically, research works on MR fluid layers can be classified as dynamic analysis and control studies. In literature, many research works have investigated the behavior of engineering structures containing MR layer under magnetic field and in case of active vibration control [10,11,12,13,14,15,16,17]. The recent studies have examined the dynamic behavior of the sandwich structures in different configurations using MR fluid as the core material [18,19,20].

In this study, the ferromagnetic feature of the MR fluid is employed for an electromagnet actuator. A flexible blade element used in a small-scale wind turbine is filled with the MR fluid and attracted by the electromagnet actuator to suppress the vibration of the flexible structure. Since the MR fluid has low stiffness effect due to its fluidity, the homogeneity and rigidity of the blade structure remains almost same by use of the MR fluid. A force relation between the electromagnet and MR fluid is derived to design a controller for active vibration control of the blade element. 

## 2. Elastic Blade Structure

**Figure 1:**
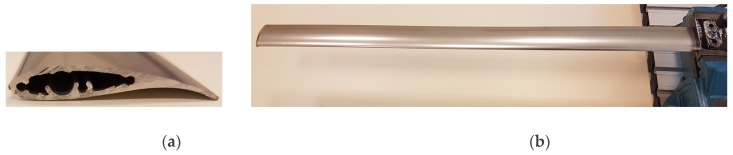
Photos of elastic blade structure (**a**) front view, (**b**) longitudinal view.

An aluminum alloy 6060 elastic blade which is used in a small-scale wind turbine test system is studied for the aims of vibration suppression. The characteristics and wind tunnel aerodynamic tests of SH3055 code numbered airfoil are reported in reference [21]. The blade structure is manufactured by a small wind turbine business company with an aluminum extrusion machine. The front and longitudinal views of the blade are shown in Figure 1a,b, respectively. As seen in Figure 1a, some portion of the airfoil is empty with extrusion roughness to reduce the weight of the blade. The blade is fixed in its one end with a clamp using a connecting piece fitted with the profile. The length of the blade after clamping is 800 mm and the width of the blade is constant in lengthwise. 

**Figure 2:**
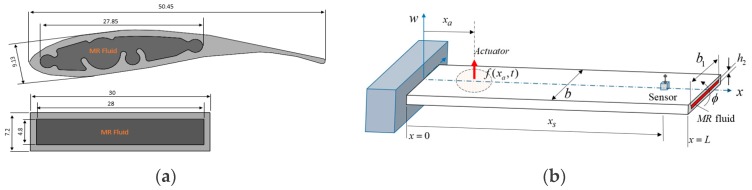
Dimensions of the blade profile (**a**) cross section (mm), (**b**) the equivalent beam model.

The cross section and main dimensions of the blade are shown in Figure 2a. The blade is filled with MR 122EG fluid (Mid-Atlantic Rubber, Inc., Baltimore, MD, USA) and both ends are closed with leak proof stick. The amount of MR fluid filled inside the blade profile is 87 mL. Since the structure of the blade is quite complicated, an equivalent beam with MR fluid is designed for modeling purposes. While the length of the equivalent beam is taken as the length of the original blade element, the width and thickness of the equivalent beam is selected so that the geometrical moment of inertia of the beam is equal to the geometrical moment of inertia of the original blade. In the designed equivalent beam, it is computed that the inner volume may contain 107.52 mL MR fluid.

### 2.1. Equivalent Beam Model

The determination of the eigenfrequencies and the corresponding eigenfunctions is necessary to analyze vibration problems in a distributed system. A modal analysis is realized for the considered elastic blade element. The equivalent beam with MR material considered in this study is schematically illustrated in Figure 2b. Hamilton principle can be used to derive the equations of motion of the elastic blade element.

(1) $$\int_{t_{1}}^{t_{2}}\left(\delta ℒ+\delta W\right)\;dt=0\;\mathrm{or}\;\delta \int_{t_{1}}^{t_{2}}\left(ℒ+W\right)\;dt=0$$

where ℒ = *T* − *V* is the Lagrangian in which *T* is the kinetic energy due to transverse and rotational motions, *V* is the corresponding potential energy due to bending stresses of the surface plates and the shear stresses of the MR layer. Also, W is the work performed by generalized forces undergoing generalized displacements. ℒ and W are given as

(2) $$\begin{array}{l} ℒ=\frac{1}{2}\left[{\int_{0}^{L}\left(\frac{\partial w(x)}{\partial t}\right)}^{2}\rho (x)\;dx+{\int_{0}^{L}\left(\frac{\partial \phi (x)}{\partial t}\right)}^{2}J(x)\;dx\right]\\ -\frac{1}{2}\left[\frac{1}{2}{\left(h_{1}+h_{2}\right)}^{2}bh_{1}E_{f}\int_{0}^{L}{\left(\frac{\partial \phi }{\partial x}\right)}^{2}dx+2E_{f}I_{f}\int_{0}^{L}{\left(\frac{\partial^{2}w}{\partial x^{2}}\right)}^{2}dx+G^{*}h_{2}b\int_{0}^{L}\gamma^{2}dx\right] \end{array}$$

(3) $$W=\int_{0}^{L}f(x,t)w(x,t)dx$$

Substituting Equations (2) and (3) into (1), the equations of motion are obtained as follow

(4) $$\rho \frac{\partial^{2}w(x)}{\partial t^{2}}+2E_{f}I_{f}\frac{\partial^{4}w(x)}{\partial x^{4}}-G^{*}b_{1}h_{2}\left(\frac{\partial^{2}w(x)}{\partial x^{2}}-\frac{\partial \phi }{\partial x}\right)=f(x,t)$$

(5) $$J\frac{\partial^{2}\phi }{\partial t^{2}}-\frac{bh_{1}E_{f}{\left(h_{1}+h_{2}\right)}^{2}}{2}\frac{\partial^{2}\phi }{\partial x^{2}}-G^{*}bh_{2}\left(\frac{\partial w}{\partial x}-\phi \right)=0$$

where w(x) is the transverse displacement of the blade and ϕ(x) is the cross-sectional rotation. Here *E_f_* is Young’s modulus, *I_f_* is the moment of inertia, *ρ* is the density of the beam cross section per unit length. Moreover, *h*_1_ is the wall thickness of the equivalent beam, *h*_2_ is the MR fluid layer thickness, *b* is the width of the beam. For the cantilever beam, the eigenfunctions are described by

(6) $$\begin{array}{l} W_{n}(x)=A_{1}\cosh\lambda_{n}x+A_{2}\sinh\lambda_{n}x+A_{3}\cos\lambda_{n}x+A_{4}\sin\lambda_{n}x\\ \Phi_{n}(x)=C_{n}(A_{1}\sinh\lambda_{n}x+A_{2}\cosh\lambda_{n}x+A_{3}\sin\lambda_{n}x+A_{4}\cos\lambda_{n}x) \end{array}$$

where *C_n_* and *λ_n_* are defined as

(7) $$C_{n}=\frac{2G^{*}h_{2}\lambda_{n}}{E_{f}h_{1}{\left(h_{1}+h_{2}\right)}^{2}\lambda_{n}{}^{2}+2G^{*}h_{2}},\;\;\lambda_{n}=\left(\frac{2n-1}{2}\pi +e_{n}\right)\frac{1}{L}$$

The vibration response of the blade element is calculated by

(8) $$w(x,t)=\sum_{n=1}^{\infty }W_{n}(x)\;e^{j\omega_{n}t},\phi (x,t)=\sum_{n=1}^{\infty }\Phi_{n}(x)\;e^{j\omega_{n}t}$$

Substituting Equations (8) into (4), the following equation is obtained as

(9) $$\sum_{n=1}^{\infty }\left[-\rho \omega_{n}^{2}W_{n}(x)+2E_{f}I_{f}\frac{d^{4}W_{n}(x)}{dx^{4}}-G^{*}bh_{2}\left(\frac{d^{2}W_{n}(x)}{dx^{2}}-\frac{d\Phi_{n}(x)}{dx}\right)\right]=0$$

Now inserting the eigenfunctions of (6) into (9), the natural frequencies of the equivalent beam containing MR fluid are calculated as follows

(10) $$\;\omega_{n}=\sqrt{\frac{2E_{f}I_{f}\lambda_{n}{}^{4}-G^{*}b_{1}h_{2}(\lambda_{n}C_{n}-\lambda_{n}{}^{2})}{\rho }}$$

where G* is the complex shear modulus of the MR material and is defined as $G^{*}=(6.125\times 10^{5}+i63773.5)$ for zero magnetic field in Reference [3]. Note that the eigenfunctions given in equations of (6) are used for uniform single material beam structures. In this study, the blade structure contains MR fluid and does not fit this condition but the effect of the MR fluid is negligible due to low stiffness effect of fluid.

### 2.2. Natural Frequency Analysis

A natural frequency analysis of the blade element was first experimentally realized using a Bruel & Kjaer-3053 vibration analyzer with Fast Fourier Transform (FFT) analysis mode. For the numerical analysis of the blade profile, a three-dimensional model of the blade was created by using computer-aided design (CAD) software SolidWorks (Waltham, MA, USA) and transferred to Ansys (Karnosboro, PA, USA) Workbench finite element software. The material properties of the MR fluid in the numerical model was taken as defined in references [22,23,24]. Using the modal analysis mode of the Ansys, the natural frequencies of the blade element model were obtained. Finally, the model of the MR fluid included equivalent beam was created and a modal analysis was also performed in the Ansys. The results of the first three natural frequencies using the defined approaches are given in Table 1. In these analysis results, it is observed that the natural frequencies obtained using different approaches are close to each other. Therefore, the equivalent beam model can be used in the model based control design study.

**Figure 3:**
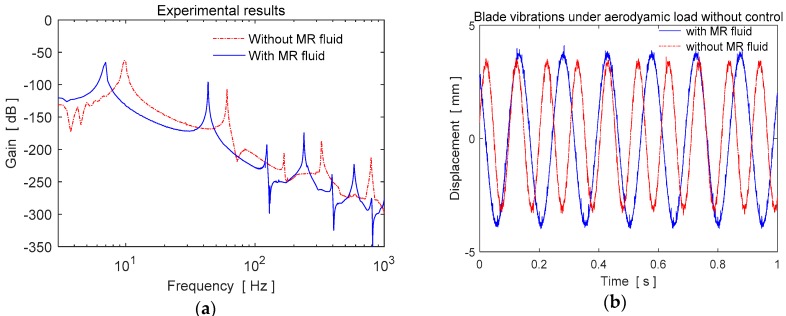
Experimental results of the empty and MR fluid filled blade without magnetic field (**a**) frequency responses, (**b**) time responses.

**Figure 4:**
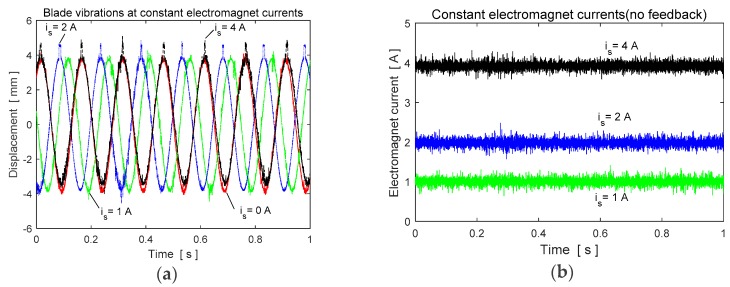
Experimental results at constant magnetic field (**a**) blade vibrations, (**b**) electromagnet currents.

To understand the effect of the MR fluid on the natural frequencies without applying a control, the experimental frequency responses of the blade element are presented in Figure 3a. As seen in Figure 3, the natural frequencies of the hollow blade element are higher than those of the MR fluid filled blade due to having less mass. The blade profile containing MR fluid is only affected by the fluid mass inside the structure. The MR fluid has no effect on the rigidity of the structure. The vibration results of the blade with and without MR fluid under an aerodynamic disturbance load without applying a control is shown in Figure 3b. The experimental blade vibrations in the case of constant electromagnet currents *i_s_* without any feedback are given in Figure 4a. The applied currents are also shown in Figure 4b. As seen in Figure 4a, the blade vibrations have some peaks at the upper side of the amplitude due to the pulling force effect of the electromagnet. 

### 2.3. State Space Model

For each vibration mode of the cantilever blade structure, the separated equation of motion is given by

(11) $${\ddot{q}}_{n}(t)+2\varsigma \omega_{n}{\dot{q}}_{n}(t)+\omega_{n}^{2}q_{n}(t)=f_{a}(t)\psi_{n}(x_{a})+f_{d}(t)\psi_{n}(x_{d})$$

where $\omega_{n}$ is the mode natural frequency, ς is the damping coefficient and $\psi_{n}(\cdot )$ is the mode shape function. Also, *f_a_* and *f_d_* are the actuator and disturbance forces respectively. The state space form for each modal equation is obtained as follows

(12) $${\dot{x}}_{n}(t)=A_{n}x_{n}(t)+B_{n}u(t)+D_{wn}d(t)$$

where $x_{n}(t)$ is the state vector, *A_n_* is the system matrix, *B_n_* is the control input matrix and u(t) is the control input. Also, d(t) shows the disturbance input. The structure of the state vector and matrices are as follows

(13) $$x_{n}=\left[\begin{array}{c} q_{n}(t)\\ {\dot{q}}_{n}(t) \end{array}\right],A_{n}=\left[\begin{array}{cc} 0 & 1\\ -\omega_{n}^{2} & -2\varsigma \omega_{n} \end{array}\right],\;B_{n}=\left[\begin{array}{c} 0\\ \psi_{n}(x_{a}) \end{array}\right],\;D_{wn}=\left[\begin{array}{c} 0\\ \psi_{n}(x_{d}) \end{array}\right]$$

If the modeling is extended for *N* modes (n=1,…,N), the state space model is given as

(14) $$\begin{array}{l} {\dot{x}}_{f}=\left[\begin{array}{c} {\dot{x}}_{1}\\ {\dot{x}}_{2}\\ \vdots \\ {\dot{x}}_{N} \end{array}\right]=\left[\begin{array}{cccc} A_{1} & & & 0\\ & A_{2} & & \\ & & \ddots & \\ 0 & & & A_{N} \end{array}\right]\left[\begin{array}{c} x_{1}\\ x_{2}\\ \vdots \\ x_{N} \end{array}\right]+\left[\begin{array}{c} B_{1}\\ B_{2}\\ \vdots \\ B_{N} \end{array}\right]u+\left[\begin{array}{c} D_{w1}\\ D_{w2}\\ \vdots \\ D_{wN} \end{array}\right]d\\ y_{f}=\left[\begin{array}{ccccc} \psi_{1}(x_{s}) & 0 & \psi_{2}(x_{s}) & 0 & \cdots \end{array}\right]\left[\begin{array}{c} x_{1}\\ x_{2}\\ \vdots \\ x_{N} \end{array}\right] \end{array}$$

The mode shape function $\psi_{n}$ for the equivalent cantilever beam is defined as

(15) $$\psi_{n}\left(x\right)=\sinh\lambda_{n}x-\sin\lambda_{n}x-\left[\frac{\sinh\lambda_{n}L+\sin\lambda_{n}L}{\cosh\lambda_{n}L+\cos\lambda_{n}L}\right]\left(\cosh\lambda_{n}x-\cos\lambda_{n}x\right)$$

A reduced order state space model for the control design study can be obtained by considering the first two modes of Equation (14). The reduced order state space equation is written as

(16) $$\begin{array}{l} {\dot{x}}_{r}(t)=A_{r}x_{r}(t)+B_{r}u(t)+D_{wr}d(t)\\ y_{r}(t)=C_{r}x_{r}(t) \end{array}$$

**Figure 5:**
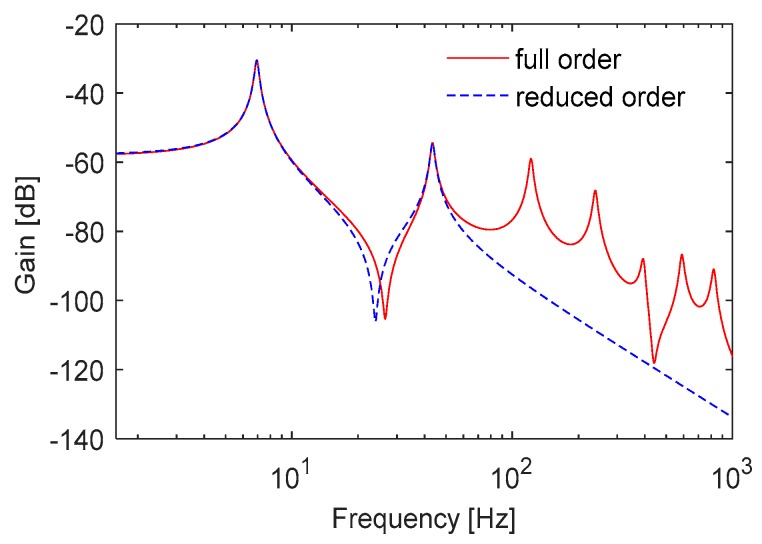
Frequency response of the blade state space models.

Distributed parameter systems have theoretically infinite number of vibration modes. The state space model derived as a full model in Equation (14) considers certain number of assumed modes. In this study, the full order model of the cantilever blade is built by considering the vibration modes up to 1 kHz. In practice, the modal contributions of the higher order modes are inconsiderable due to small modal amplitudes. Also, the reduced order model which contains the first two modes up to 60 Hz is used for controller design. The frequency responses of the full and reduced order state space models are shown in Figure 5.

### 2.4. Force Characterization

**Figure 6:**
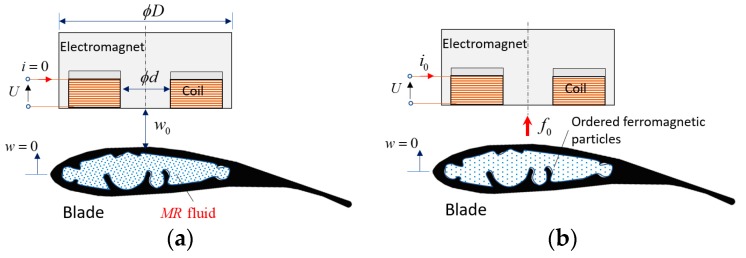

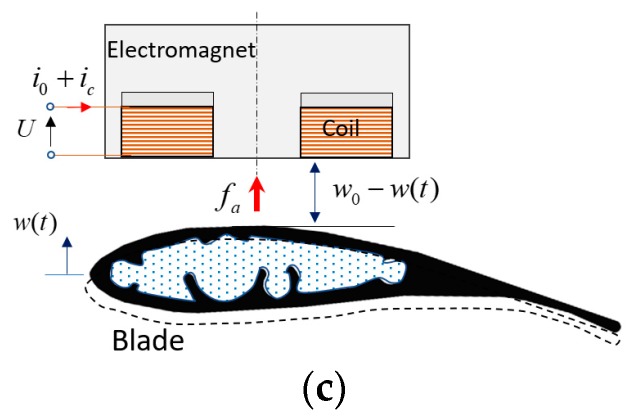
Illustration of the MR blade and electromagnetic actuator interaction (**a**) initial setting, (**b**) the application of the bias current *i*_0_, (**c**) the control force *f_a_* generation.

The interaction between the electromagnet and the MR fluid should be modeled for a control design study. To understand the interaction phenomenon cross section views are illustrated in Figure 6a,c. The electromagnet is positioned over the MR blade element with an air gap (Figure 6a). When the current is supplied to the electromagnet and a magnetic field is generated, the iron particles in the MR fluid become ordered and an attractive force is applied on the blade (Figure 6b). At this stage, the blade stands at the nominal position and still not moving. When the current increases the pulling force also increases and the blade moves to the actuator side as shown in Figure 6c. The magnitude of the force depends on the magnetic flux given by the controller according to the displacement information of the blade obtained using an optical sensor in a feedback control structure. The nonlinear magnetic force is derived as

(17) $$f_{m}(t)=k\frac{{\left(i_{0}+i_{c}(t)\right)}^{2}}{{\left[w_{0}-w(t)\right]}^{2}}$$

where $k=1/4\mu_{0}N^{2}A$. Here *A* is the area of the electromagnet, *N* is the number of the coil turn and $\mu_{0}$ is the vacuum permeability. Also, $i_{c}$ is the control current. The electromagnetic force is linearized around $\left(i_{0},w_{0}\right)$ values as follows.

(18) $$f_{a}(t)=K_{w}w+K_{i}i_{c}$$

where $K_{w}=2k(i_{0}^{2}/w_{0}^{3})$ and $K_{i}=2k(i_{0}/w_{0}^{2})$. 

**Figure 7:**
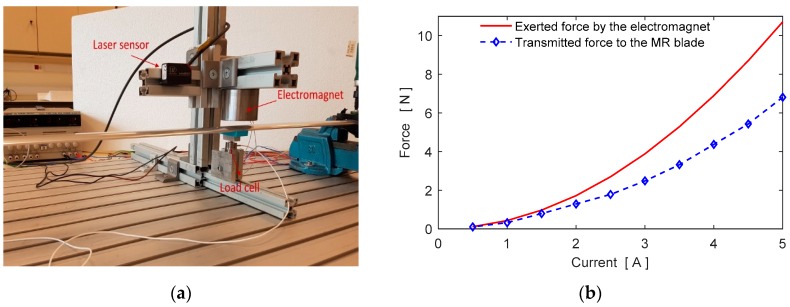
Force measurements (**a**) Experimental setup, (**b**) Variation of the electromagnetic force and the transmitted force.

It is important to show how much electromagnetic force is transmitted to the MR blade. To understand the force variation at the MR blade side a load cell is installed under the blade as shown in Figure 7a. In experiments, the air gap between the electromagnet and MR blade is set to 1 mm and the coil current is increased 1 A for each step and the force generated on the blade is measured by the load cell. It is not possible to measure the electromagnetic force directly but the magnetic field can be measured by a gauss meter. The electromagnetic force is computed using magnetic field measurements with the equation $f_{e}=B^{2}(A/\mu_{0})$. Here, *B* is the magnetic field measured by using a gauss meter. The variations of the experimental forces are shown in Figure 7b. At large coil currents, the loss is increasing as seen in Figure 7b.

**Figure 8:**
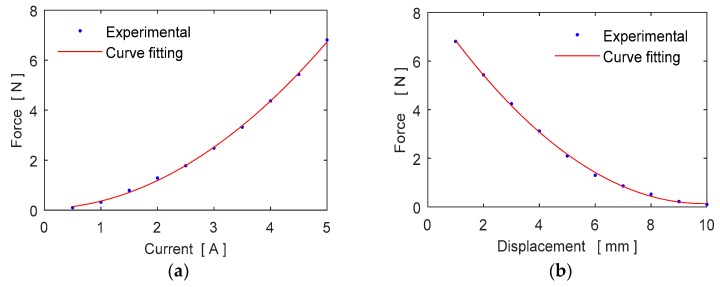
Variation of the electromagnetic force (**a**) with current, (**b**) with displacement.

Variations of the electromagnetic force with the coil current and the gap are also analyzed. The coil current of the electromagnet is increased from 0.5 amperes to 5 amperes by setting the electromagnet at a fixed distance of 1 mm from the blade surface. Similarly, while the coil current set to 5 amperes, the gap between the electromagnet and the blade surface is increased by 1 mm for each measurement. Figure 8a,b shows the variation of electromagnetic response force with the coil current and the displacement respectively. As seen in these figures the characteristic of the force is nonlinear with respect to the displacement and current. 

### 2.5. Aerodynamic Load

**Figure 9:**
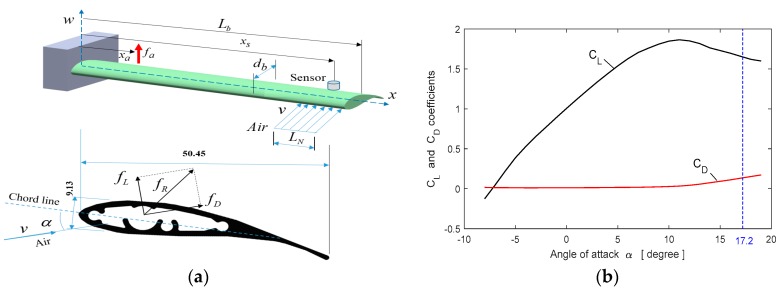
Aerodynamic load (**a**) the blade layout, (**b**) variation of the lift and drag coefficients.

To test the designed controller under a steady state disturbance, an aerodynamic load effecting on the blade structure is created using an air nozzle. In the case of wind turbines, the air current flows over the wings with a certain angle of attack. Lift and drag forces occur as the air passes over the blades and drag force causes vibrations on the blade. These forces are defined as

(19) $$f_{L}=\frac{1}{2}C_{L}\rho L_{N}d_{b}v^{2}\;,\;f_{D}=\frac{1}{2}C_{D}\rho L_{N}d_{b}v^{2}$$

where *C_L_* and *C_D_* are lift and drag coefficients. Also, *L_N_* is the air load length, *d_b_* is the width of the blade and *ν* is the relative wind speed. The wing is forced to bend by the effect of aerodynamic drag force *f_D_*. The lift and drag coefficient data of the SH3055 airfoil used in the blade element of this study is given in reference [25]. The layout of the blade and the variation of the lift and drag coefficients are shown in Figure 9. To create an aerodynamic load in the experimental system, the air nozzle blows air to the end of the blade element in adjusted air speeds. In the experimental system, it is observed that the blade starts to vibrate when the angle of attack of the nozzle is 14 degrees due to increasing of the drag force on the blade. In this study, the angle of attack of the air nozzle is set to 17.2 degree.

## 3. Control Principle

**Figure 10:**
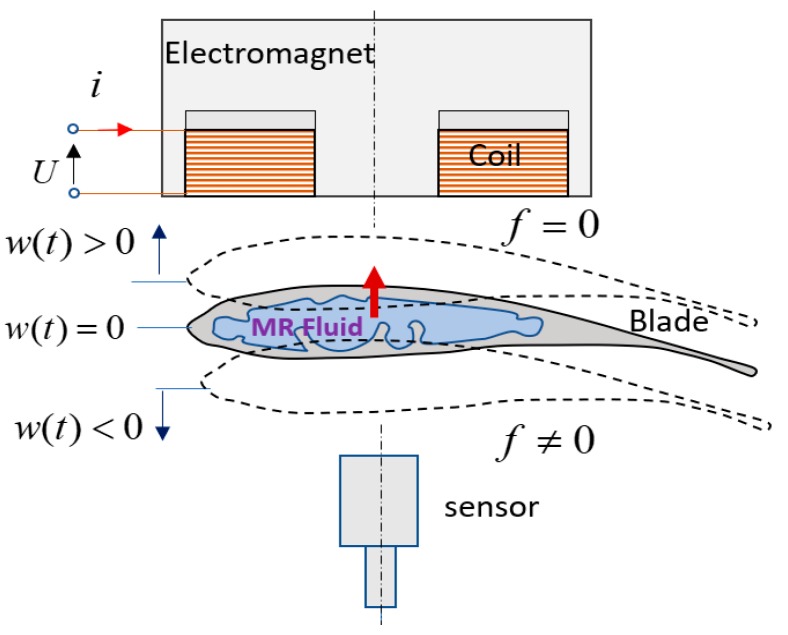
Control principle of the blade vibrations.

To suppress the vibration of the blade element the electromagnetic actuator applies an attractive force to the MR blade according to blade position measured by a sensor. In this study, the magnetic field or resulting magnetic force of the electromagnet is controlled by driving the coil current. Since only one actuator is employed, the attractive force is generated when the blade moves to downward from the nominal position. Otherwise, the electromagnet does not generate the attractive force when the blade approaches to the electromagnet. Control principle of the blade vibrations is illustrated in Figure 10. 

### 3.1. Multi-Objective H_2_/H_∞_ Control

**Figure 11:**
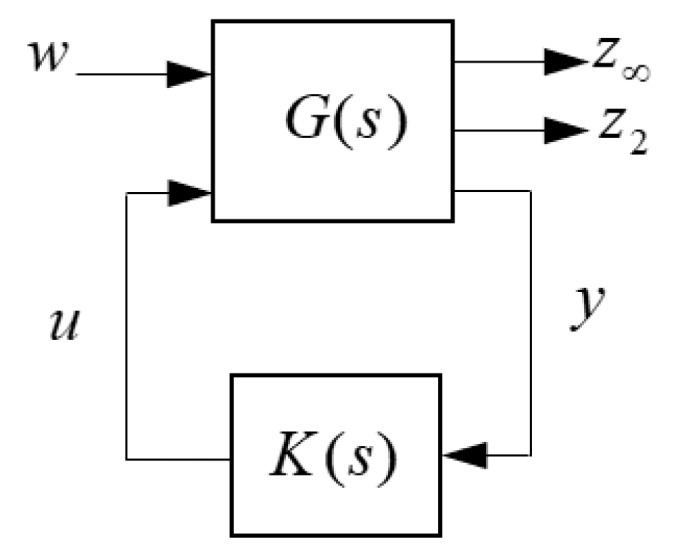
Multi-objective control structure.

Norm based robust control approaches are suitable for the distributed parameter and structural systems due to considering unmodelled high frequency modal dynamics in control design [26]. Therefore, robust controllers can avoid spillover effect in flexible systems. In control design studies, *H*_2_ and *H*_∞_ norm performances are two important specifications. While *H*_∞_ performance is convenient to enforce robustness against model uncertainty, *H*_2_ performance is useful to handle stochastic aspects such as measurement noise and control cost. The generalized control design block structure of the multi-objective control is shown in Figure 11. The output channel *z*_∞_ is associated with the *H*_∞_ performance while the channel *z*_2_ is associated with the *H*_2_ performance. Also, *T*_∞_(*s*) and *T*_2_(*s*) are the closed-loop transfer functions from w to *z*_∞_ and *z*_2_, respectively. The state-space realization of the augmented plant having both design objectives is given by

(20) $$\begin{array}{l} \dot{x}=Ax+B_{1}w+B_{2}u\\ z_{\infty }=C_{\infty }x+D_{\infty 1}w+D_{\infty 2}u\\ z_{2}=C_{2}x+D_{21}w+D_{22}u\\ y=C_{y}x+D_{y1}w \end{array}$$

Using the closed loop transfer functions, minimization of a trade-off criterion can be formed such that design a controller *K*(*s*) that minimizes the mixed *H*_2_/*H*_∞_ norm criterion

(21) $$\alpha {\left\|T_{\infty }(s)\right\|}_{\infty }^{2}+\beta {\left\|T_{2}(s)\right\|}_{2}^{2}\alpha ,\beta \geq 0$$

subject to

(22) $${\left\|T_{\infty }(s)\right\|}_{\infty }^{}<\gamma_{0},{\left\|T_{2}(s)\right\|}_{2}^{}<\nu_{0},\gamma_{0},\nu_{0}>0$$

**Figure 12:**
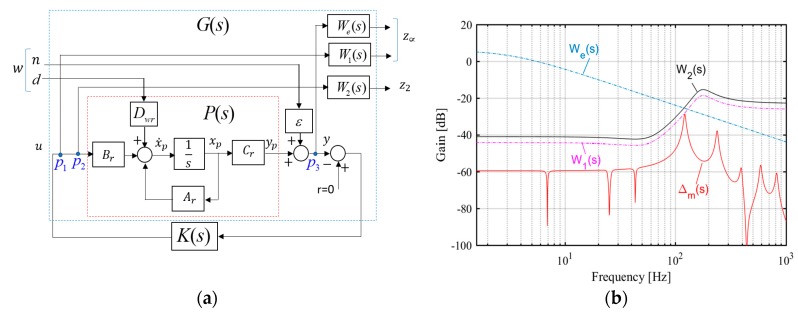
Multi-objective control design approach (**a**) the block structure, (**b**) frequency responses of the weighting filters and uncertainty.

The control design block structure is shown in Figure 12a. In this block, *P*(*s*) is the reduced order plant model and *K*(*s*) is the controller that will be designed. In the blade system, the input *η* shows the aerodynamic disturbances and sensor noise. Also, the input *d* represents the disturbance caused by unstructured uncertainty or unmodeled high frequency dynamics in the system. The controlled variables *z*_∞_ and *z*_2_ are outputs of the transfer functions obtained for each external input. Consider the points *p*_1_, *p*_2_ and *p*_3_ in Figure 12a. The transfer functions from *η* to *p*_1_, *p*_2_ and *p*_3_ are obtained as

(23) $$\begin{array}{l} G_{p_{1}n}(s)=-\frac{K(s)\varepsilon }{I+P(s)K(s)}=-\varepsilon K(s)S(s),G_{p_{3}n}(s)=\frac{\varepsilon }{I+P(s)K(s)}=\varepsilon S(s)\\ G_{p_{2}n}(s)=G_{p_{1}n}(s) \end{array}$$

where *S*(*s*) = (*I* + *P*(*s*)*K*(*s*))^−1^ is the sensitivity transfer function. Moreover, the transfer functions from *d* to the points *p*_1_, *p*_2_ and *p*_3_ are given as

(24) $$\begin{array}{l} G_{p_{1}d}(s)=-\frac{P(s)K(s)}{I+P(s)K(s)}=-T(s),G_{p_{3}d}(s)=\frac{P(s)}{I+P(s)K(s)}=T_{c}(s)\\ G_{p_{2}d}(s)=G_{p_{1}d}(s)\; \end{array}$$

Here *T*(*s*) is the complimentary sensitivity transfer function and *T_c_*(*s*) is the settling function. In a control system design, shaping with *T*(*s*) transfer function is preferable for noise attenuation and tracking. Also, *T*(*s*) transfer function is important for robust stability with respect to multiplicative uncertainties at the system output. In control block structure, the control cost is adjusted both *H*_2_ and *H*_∞_ norm objectives. The weighting function *W*_2_(*s*) is used to compromise between the control effort and the disturbance rejection performance. The weighting functions for control design are selected as

(25) $$W_{1}(s)=\alpha_{1}\times \frac{s^{2}+2\omega_{nm}\varsigma_{nm}+\omega_{nm}{}^{2}}{s^{2}+2\omega_{dm}\varsigma_{dm}+\omega_{dm}{}^{2}},W_{2}(s)=\alpha_{2}\times \frac{s^{2}+2\omega_{nm}\varsigma_{nm}+\omega_{nm}{}^{2}}{s^{2}+2\omega_{dm}\varsigma_{dm}+\omega_{dm}{}^{2}},W_{e}(s)=\alpha_{e}\times \frac{\sigma }{s+\omega_{e}}$$

The frequency shaping filters *W*_1_(*s*) and *W*_2_(*s*) are selected in a systematic rule using multiplicative uncertainty which represents unmodeled high-frequency vibration mode dynamics in the control system model [27]. While the numerator frequency $\omega_{nm}$ of the filter is taken as the controlled last vibration mode frequency, the denominator frequency $\omega_{dm}$ is selected as the first unmodeled frequency or the third vibration mode frequency. The multiplicative uncertainty $\Delta_{m}(j\omega )$ in the system is obtained as

(26) $$\Delta_{m}(j\omega )=\frac{P_{f}(j\omega )-P_{r}(j\omega )}{P_{r}(j\omega )}$$

where $P_{f}(j\omega )$ and $P_{r}(j\omega )$ show the full and reduced order system model, respectively. The robust stability filter *W*_1_(*s*) essentially covers the unstructured uncertainties existing in the system such as

(27) $$\left|\Delta_{m}(j\omega )\right|\leq \left|W_{1}(j\omega )\right|\forall \omega ,$$

The frequency responses of the filters and multiplicative uncertainty are shown in Figure 12b. Using the transfer functions obtained in Equations (24) and (25), the controlled outputs with *H*_∞_ norm objective are derived as $z_{\infty }^{\left(1\right)}=W_{1}(s)G_{p_{1}n}(s)$, $z_{\infty }^{\left(2\right)}=W_{e}(s)G_{p_{3}n}(s)$,$z_{\infty }^{\left(3\right)}=W_{1}(s)G_{p_{1}d}(s)$ and $z_{\infty }^{\left(4\right)}=W_{e}(s)G_{p_{3}d}(s)$. Also, the outputs with $H_{2}$ norm objective are obtained as $z_{2}^{\left(1\right)}=W_{2}(s)G_{p_{2}n}(s)$ and $z_{2}^{\left(2\right)}=W_{2}(s)G_{p_{2}d}(s)$. In robust control design, mixed-sensitivity approach shapes one or more closed loop transfer functions [28]. The objective of the control design configuration is to minimize

(28) $${\left\|\left[\begin{array}{c} W_{e}(s)\varepsilon S(s)\\ W_{1}(s)\varepsilon K(s)S(s)\\ W_{e}(s)T_{c}(s)\\ W_{1}(s)T(s) \end{array}\right]\right\|}_{\infty }<\gamma_{0}\;\mathrm{and}\;{\left\|\left[\begin{array}{c} W_{2}(s)\varepsilon K(s)S(s)\\ W_{2}(s)T(s) \end{array}\right]\right\|}_{2}<\nu_{0}$$

### 3.2. Controller Design

**Figure 13:**
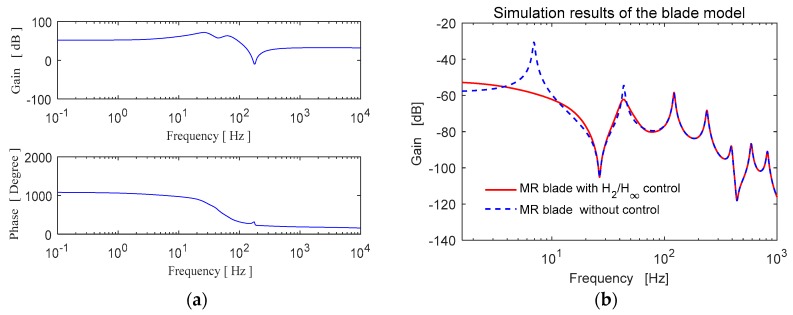
Frequency responses (**a**) the multi-objective controller, (**b**) the closed-loop system.

The multi-objective *H*_2_/*H*_∞_ controller is designed using *hinfmix* command in Matlab (MathWorks, Natick, MA, USA). This function performs multi-objective output-feedback synthesis and computes a linear time-invariant (LTI) controller that minimizes the mixed *H*_2_/*H*_∞_ norm criterion. The frequency response characteristic of the *H*_2_/*H*_∞_ controller is shown in Figure 13a. The closed loop frequency response is given in Figure 13b. 

## 4. Experimental System

**Figure 14:**
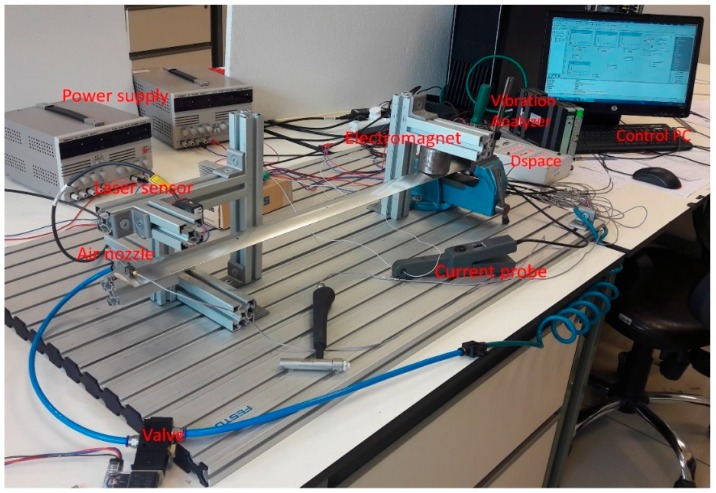
Experimental system setup.

The photo of the experimental system setup is shown in Figure 14. The blade test system explained in Section 2 is studied for vibration suppression. In the experimental setup, an Advanced Motion Controls MC1XAZ01 (Servo Systems Co., Rockaway, NJ, USA) current driver with 0–17 A range is used to drive the electromagnetic actuator. Power supplies, optic sensor and Quanser-Q8 (Quanser, Markham, Canada) unit are used as peripheral devices. Vibration analysis of the blade is performed with a Bruel & Kjaer 3053 device (Nairum, Denmark). The designed multi-objective controller is implemented using a dSpace 1104 control card (dSPACE GmbH, Paderborn, Germany). The controller is discretized and compiled in the state space form using a Matlab/Simulink file and installed on a dSpace control card.

### Experimental Results

**Figure 15:**
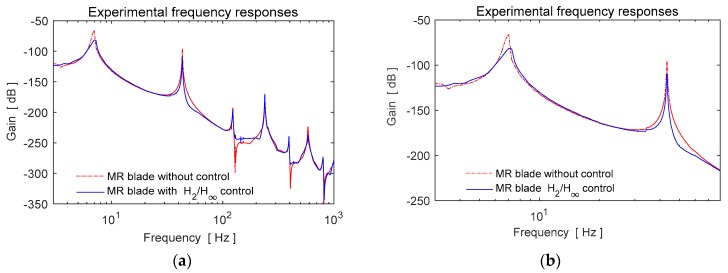
Experimental frequency responses of the closed loop (**a**) all modes, (**b**) first two modes.

The closed loop frequency responses of the MR fluid blade element obtained in experiments with *H*_2_/*H*_∞_ controller is shown in Figure 15. Since the first two modes of the cantilever blade are targeted in control design, these modes are suppressed by the controller in different levels of gains as shown in Table 2. As seen in Figure 15a, the uncontrolled high frequency vibration modes are not excited by the controller. 

**Figure 16:**
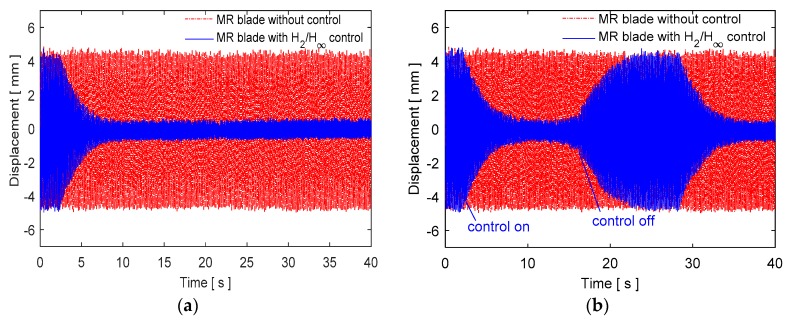
Experimental results of the closed loop system under steady state aerodynamic load (**a**) continuous control, (**b**) repeated control.

The designed multi-objective controller is tested in different conditions under the steady state aerodynamic load. Experimental time history responses of the closed loop system for a continuous control case from a starting time are shown in Figure 16a. Moreover, the repeated controlled and uncontrolled tests are realized to understand the response characteristics of the controllers as given in Figure 16b. 

**Figure 17:**
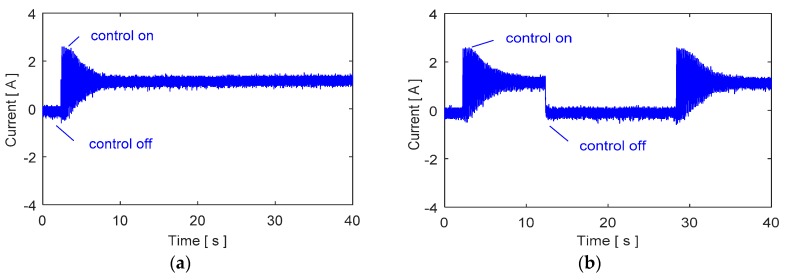
Experimental control inputs (**a**) continuous control, (**b**) repeated control.

**Figure 18:**
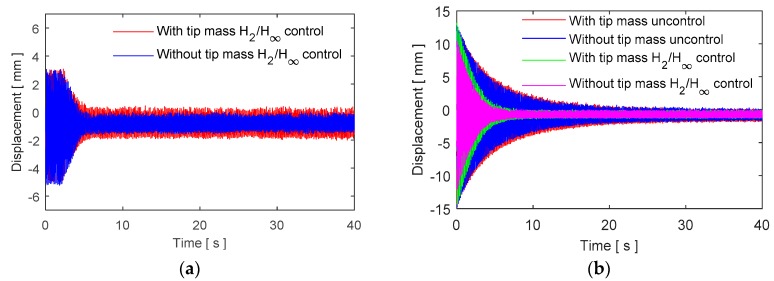
Robustness test results for an additional mass, (**a**) steady state case, (**b**) reference point case.

The control inputs are shown in Figure 17 for the control case of Figure 16. Moreover, a robustness test is performed in the case of parameter variation by attaching an extra mass on the surface of the blade. The results are shown in Figure 18 when an additional mass of 12% of the blade mass is added. The control system is quite robust against parameter uncertainty and vibration attenuation with additional mass.

## 5. Conclusions

In this research study, vibration of a small-scale wind turbine blade was suppressed using a MR fluid-electromagnetic actuator under the effect of a steady state aerodynamic disturbance. A force based interaction model between the MR blade and the electromagnet was derived and some characterization works were presented. A mixed *H*_2_/*H*_∞_ controller was designed to attenuate the vibration of the blade structure. Some experiments were realized to show the effectiveness of the proposed MR blade electromagnetic actuator for the transient and steady state aerodynamic loads. The experimental frequency response and the time history of the closed loop system showed significant vibration reduction in the blade element. The application of the proposed MR fluid-electromagnetic actuator may be possible for the small and sub-medium size wind turbine blades. The electromagnets should move with the blades during wind turbine operation by using slip rings to conduct power to the electromagnets. Moreover, a local application of the MR layer on the blade can be developed and it is not necessary to fill whole blade with MR fluid. 

## Figures and Tables

**Table 1:** Natural frequencies of the blade profile and equivalent beam.

Natural Frequencies	Without MR Fluid-Blade Profile Experimental Results [Hz]	Blade Profile Experimental Results [Hz]	Blade Profile ANSYS Results [Hz]	Equivalent Beam Element ANSYS Results [Hz]	Equivalent Beam Analytical Model [Hz]
1	9.5	7	7.44	7.24	7.15
2	59.5	43.50	46.25	45.308	44.81
3	167	123.5	127.12	125.74	125.48

**Table 2:** Amount of reduction in gains with *H*_2_/*H*_∞_ controller.

Mode Number	Gain Reduction [dB]
1.mode	15.83
2.mode	13.07

## References

[1] Sun Q., Zhou J.X., Zhang L. (2003). An adaptive beam model and dynamic characteristics of magnetorheological materials. J. Sound Vib..

[2] Yang G. (2001). Large-Scale Magnetorheological Fluid Damper for Vibration Mitigation: Modeling, Testing and Control. Ph.D. Thesis.

[3] Yalcintas M., Dai H. (1999). Magnetorheological and electrorheological materials in adaptive structures and their performance comparison. Smart Mater. Struct..

[4] Spencer B.F., Nagarajaiah S. (2003). State of the art of structural control. J. Struct. Eng..

[5] Chen L., Hansen C.H. Active vibration control of a magnetorheological sandwich beam. Proceedings of the Australian Acoustical Society Conference.

[6] Xu Y.L., Qu W.L., Ko J.M. (2000). Seismic response control of frame structures using magnetorheological/electrorheological dampers. Earthq. Eng. Struct. Dyn..

[7] Stanway R., Sproston J.L., El Wahed A.K. (1996). Applications of electrorheological fluids in vibration control: A survey. Smart Mater. Struct..

[8] Niu H., Zhang Y., Zhang X., Xie S. (2010). Active vibration control of plates using electro-magnetic constrained layer damping. Int. J. Appl. Electromagn. Mech..

[9] Valevate A.V. (2004). Semi-Active Vibration Control of a Beam Using Embedded Magneto-Rheological Fluids. Ph.D. Thesis.

[10] Choi S.B., Park Y.K., Jung S.B. (1999). Modal characteristics of a flexible smart plate filled with electrorheological fluids. J. Air..

[11] Choi S.B., Thompson B.S., Gandhi M.V. (1995). Experimental control of a single-link flexible arm incorporating electrorheological fluids. J. Guid. Control Dyn..

[12] Rajamohan V., Sedaghati R., Rakheja S. (2011). Optimal vibration control of beams with total and partial MR-fluid treatments. Smart Mater. Struct..

[13] Yeh J.Y. (2013). Vibration analysis of sandwich rectangular plates with magnetorheological elastomer damping treatment. Smart Mater. Struct..

[14] Cortés F., Sarría I. (2015). Dynamic analysis of three-layer sandwich beams with thick viscoelastic damping core for finite element applications. Shock Vib..

[15] Martin L.A. (2011). A Novel Material Modulus Functions for Modeling Viscoelastic Materials. Ph.D. Thesis.

[16] Hirunyapruk C. (2009). Vibration Control Using an Adaptive Tuned Magneto-Rheological Fluid Vibration Absorber. Ph.D. Thesis.

[17] Hu B., Wang D., Xia P., Shi Q. (2006). Investigation on the vibration characteristics of a sandwich beam with smart composites-MRF. World J. Model. Simul..

[18] Kolekar S., Venkatesh K., Oh J.S., Choi S.B. (2017). The tenability of vibration parameters of a sandwich beam featuring controllable core: Experimental Investigation. Adv. Acoust. Vib..

[19] Malaeke H., Moeenfard H., Ghasemi A.H., Baqersad J. (2017). Vibration suppression of MR sandwich beams based on fuzzy logic. Shock & Vibration, Aircraft/Aerospace, Energy Harvesting, Acoustics & Optics.

[20] Manoharan R., Vasudevan R., Sudhagar P.E. (2016). Semi-active vibration control of laminated composite sandwich plate—An experimental study. Arch. Mech. Eng..

[21] Somers D.M., Maughmer M.D. (2003). Theoretical Aerodynamic Analyses of Six Airfoils for Use on Small Wind Turbines.

[22] Eshaghi M., Rakheja S., Sedaghati R. (2015). An accurate technique for pre-yield characterization of MR fluids. Smart Mater. Struct..

[23] Fonseca H.A., Gonzalez E., Restrepo J., Parra C.A., Ortiz C. (2016). Magnetic effect in viscosity of magnetorheological fluids. J. Phys. Conf. Ser..

[24] Weiss K.D., Carlson J.D., Nixon D.A. (1994). Viscoelastic properties of magneto-and electro-rheological fluids. J. Intell. Mater. Syst. Struct..

[25] Selig M.S., McGranahan B.D. (2004). Wind tunnel aerodynamic tests of six airfoils for use on small wind turbines. J. Sol. Energy Eng. (Trans. ASME).

[26] Sivrioglu S., Nonami K. (1997). Active vibration control by means of LMI-based mixed H_2_/H_∞_ state feedback control. JSME Int. J. Ser. C Mech. Syst. Mach. Elem. Manuf..

[27] Sivrioglu S., Tanaka N., Yuksek I. (2002). Acoustic power suppression of a panel structure using H-infinity output feedback control. J. Sound Vib..

[28] Zhou K., Doyle J.C. (1997). Essentials of Robust Control.

